# The Wnt Antagonist sFRP1 as a Favorable Prognosticator in Human Biliary Tract Carcinoma

**DOI:** 10.1371/journal.pone.0090308

**Published:** 2014-03-03

**Authors:** Pengcheng Kang, Ming Wan, Peng Huang, Chunlong Li, Zhidong Wang, Xiangyu Zhong, Zhanliang Hu, Sheng Tai, Yunfu Cui

**Affiliations:** Department of Hepatopancreatobiliary Surgery, Second Affiliated Hospital of Harbin Medical University, Harbin, P.R. China; University of Nebraska Medical Center, United States of America

## Abstract

Inactivation of Secreted Frizzled-Related Protein-1 (SFRP1) and overexpression of β-catenin play important roles in the development and progression of a wide range of malignancies. We sought to determine whether the expression of SFRP1 and β-catenin correlates with clinicopathologic parameters in human biliary tract cancer (BTC) and to evaluate the potential roles of these proteins as prognostic indicators. The expression of SFRP1 and β-catenin in 78 patients with BTC and 36 control patients as investigated by immunohistochemistry. A wide variety of statistical parameters were assessed to determine the association between these proteins and the occurrence, clinical features, and overall survival rate in BTC.SFRP1 and β-catenin had an inverse correlation (r = −0.636, P<0.0001) as assessed by Spearman rank analysis, with 52 (66.7%) of the BTC samples negative for SFRP1 expression and 53 (68.0%) positive for β-catenin expression. Expression of each protein was associated with the histological type and lymph node invasion of BTC. A significantly poorer overall survival rate was observed for patients with low SFRP1 expression (P<0.0001) or high β-catenin expression (P = 0.007). SFRP1 expression (P<0.0001), β-catenin expression (P<0.01) and histological type (P<0.01) were correlated with overall survival rate as assessed by univariate analysis; while multivariate analysis suggested that SFRP1 (hazard ratio, 10.514; 95% confidence intervals, 2.381–39.048; P<0.0001) may serve as an independent prognostic factor for BTC. Collectively, these results demonstrate that SFRP1 is a favorable prognostic factor for human BTC and that its expression inversely correlates with that of β-catenin.

## Introduction

Biliary tract cancer (BTC), which is the second most common primary hepatobiliary malignancy, arises from the ductal epithelium of the biliary tree and is characterized by increasing incidence and poor prognosis [1]–[3]. At present, radical surgical resection, chemotherapy or radiation are used as curative treatments for BTC that is diagnosed in early stages. However, this disease is usually fatal, with most patients diagnosed in advanced stages [1], [4], [5]. Therefore, it is imperative to find effective biomarkers for early diagnosis and to elucidate the molecular mechanisms associated with pathogenesis [6]–[7].

The Wnt signaling pathway plays an important role in embryonic development, cell differentiation and cell proliferation [8], and its role in bile duct development has been established [9]. Wnt proteins are secreted molecules that interact with transmembrane receptors of the frizzled family. β-catenin is the most important component of the Wnt signaling pathway, with its altered localization serving as a marker of pathway activation. Once the Wnt ligand binds to its receptor, a complex signaling cascade is activated that leads to stabilization of cytosolic β-catenin. The β-catenin then pools and translocates to the nucleus, where it interacts with LEF/TCF transcription factor, leading to the activation of downstream target genes [10], including several oncogenes, such as c-myc, cyclin D1 and c-jun [11]–[12]. Recently, the aberrant activation of the Wnt signal transduction pathway has been shown to be closely linked to tumorigenesis in adults [13]–[15].

Secreted frizzled-related protein-1 (SFRP1) is an antagonist of the Wnt signaling pathway that maps to chromosome 8p12-p11.1. SFRP1 harbors a domain that is homologous to the frizzled receptors [16] and can bind Wnt proteins in the extracellular compartment, thus inhibiting ligand–receptor interaction and signal transduction[17]. Inactivation of SFRP1 has been reported in a wide range of human malignancies, including ovarian [18], bladder [19], mesothelioma [20], breast [21]–[22], esophageal [23], prostate [24]and gastric cancers [25]. Furthermore the SFRP1 gene displays a high methylation frequency in cholangiocarcinoma, suggesting the possibility of its tumor-specific inactivation [26]. However, to our knowledge, the role of SFRP1 expression in human biliary tract carcinoma has not been directly examined.

The purpose of this study was to examine SFRP1 and β-catenin expression in human BTC by immunohistochemistry. We evaluated the relationship of their expression with clinicopathologic parameters. Our results suggest that SFRP1 may serve as a candidate tumor suppressor gene for BTC.

### Patients and Methods

We obtained a total of 114 samples from the Second Affiliated Hospital of Harbin Medical University, Heilongjiang, China from April 2008 to June 2010, including 78 samples from patients with pathologically confirmed BTC and 36 normal bile duct samples. Among the tumor samples, 34 were males and 44 females, with a median age of 57.6 years, ranging from 37 to 78 years.

Normal bile ducts were collected from patients undergoing pancreatoduodenectomy for treatment of pancreatic or duodenal nontumor diseases, while their bile ducts remained diseases-free. The median age of the control group was 52.4 years, ranging from 42 to 73 years. Samples were collected according to the approval of the ethical committee of the Second Affiliated Hospital of Harbin Medical University, with written individual consent from each patient for this study. The pathological data of all patients that received curative resection were assessed based on the 7th edition of the American Joint Committee on Cancer for BTC. All patients were followed up to June 2013.

### Immunohistochemistry

Surgical specimens were stained with hematoxylin-eosin to identify a representative paraffin block with typical tumor morphology. We used successive paraffin-embedded sections at 4 µm intervals for assessment. All sections were deparaffinized with xylene and rehydrated with graded ethanol solution. The sections were then incubated in 3% hydrogen peroxide at room temperature for 15 min to inactivate endogenous peroxidases. After washing with phosphate-buffered saline, antigen retrieval was performed in citrate buffer (pH  = 6.0) for 20 min in a microwave oven to enhance the immunoreactivity. Then the sections were incubated in blocking serum for 30 min at 37°C to reduce nonspecific binding, followed by incubation with anti-SFRP1 rabbit polyclonal antibody (Abgent, SanDiego, USA) or anti-β-catenin mouse monoclonal antibody (Santa Cruz, Biotechnology, Inc) at a dilution of 1∶100 overnight at 4°C. As a negative control, tissue sections were incubated with phosphate-buffered saline instead of the primary antibodies. The sections were incubated with horseradish peroxidase (HRP)–streptavidin complex for 30 min. Then, biotinylated antirabbit immunoglobulin or goat antimouse secondary antibody and streptavidin peroxidase complex reagent were applied. All sections were counterstained with hematoxylin after visualization with the diaminobenzidine chromogen.

Positive cases of staining for SFRP-1 exhibited clear cytoplasmic staining that was predominately perinuclear. Positive cases for β-catenin staining exhibited cytomembrane and nuclear staining. Scoring of the staining was according to the following criteria: (−), no cell staining; (+), <25% cell staining; (++), 25–50% cell staining; and (+++), >50% cell staining. For all cases, (−) and (+) were defined as negative expression, and (++) and (+++) as positive expressions. Each stained section was blindly evaluated by two investigators unaware of the clinical information. Samples were observed under high power magnification (200×).

### Statistical analysis

Statistical analyses were performed with SPSS 17.0 software. Chi-square tests were used to clarify the relationship between SFRP1/β-catenin expression and clinicopathologic features. Overall patient survival rates were calculated using the Kaplan-Meier method, with patient survival times calculated from the time of surgery to the time of death or the most recent follow-up. Spearman rank correlation was carried out to evaluate the relationship between SFRP1 and β-catenin expression. The significance of clinicopathologic parameters was determined by univariate log-rank testing, and the Cox proportional hazards regression model was applied to analyze independent prognostic factors selected by univariate analysis. P<0.05 was considered statistically significant.

## Results

### Patient information

A total of 78 BTC patients were registered in this study, including 34 males and 44 females with an average age of 57.5 years (Table 1, first two columns). According to the American Joint Committee on Cancer classification, 10 were stage I patients, 24 stage II patients, 16 stage III patients and 28 stage IV patients. The clinical characteristics of the patients, including TNM staging, histological type, lymph node invasion, differentiation grade, metastasis after surgery and 3-year survival, are shown, as well as serum CEA and CA-19-9 levels and HBV infection status before and after surgery. The follow-up period (the time from the operation date to the day of the last visit) continued until June 2013. The survival time ranged from 1 to 36 months (mean 23.3 months). During this period, 46 patients died of recurrence.

**Table 1:** Relationships between the expression of SFRP1, β-catenin and clinicopathologic features in human biliary tract cancer.

Clinicopathologic features							
	n	SFRP1		*P*	β-catenin		*P*
		Negative n = 52	Positive n = 26		Negative	Positive	
**Gender**							
Male	34	20	14	0.196	10	24	0.661
Female	44	32	12		15	29	
**Age (years)**							
<60	32	22	10		8	24	0.266
≥60	46	30	16	0.745	17	29	
**TNM stage**							
I	10	7	3	0.182	4	6	**0.039**
II	24	18	6		4	20	
III	16	7	9		3	13	
IVa	28	20	8		14	14	
**Histological type**							
Adenocarcinoma	70	44	26	**0.01**	24	46	**0.05**
Mcinous adenocarcinoma	5	5	0		1	4	
Papillary carcinoma	3	3	0		0	3	
**Anatomical distribution**							
Intrahepatic	20	14	6	0.599	5	15	0.233
Perihilar	31	22	9		8	23	
Distal	27	16	11		12	15	
**Lymph node invasion**							
Present	47	36	11	**0.022**	5	37	**0.012**
Absent	31	16	15		12	16	
**Differentiation grade**							
Well/moderately	36	20	16	0.054	12	24	0.822
Poorly/undifferentiated	42	32	10		13	29	
**Metastasis after surgery**							
+	44	28	16	0.518	14	30	0.960
−	34	24	10		11	23	
**Survival rate**							
>3 years	32	16	16	**0.009**	15	17	**0.019**
≤3 years	46	36	10		10	36	
**Serum CEA level**							
>5 ng/ml	45	27	18	0.145	14	31	0.835
≤5 ng/ml	33	25	8		11	22	
**Serum CA19-9 level**							
>37 U/ml	48	31	17	0.622	15	33	0.848
≤37 U/ml	30	21	9		10	20	
**HBV infection**							
+	40	25	15	0.423	13	27	0.931
−	38	27	11		12	26	
							

As a control, we also collected 36 normal bile duct samples from patients undergoing pancreatoduodenectomy for treatment of non-cancerous pancreatic or duodenal disease.

### SFRP1 expression is reduced and β-catenin expression is increased in BTC

**Figure 1:**
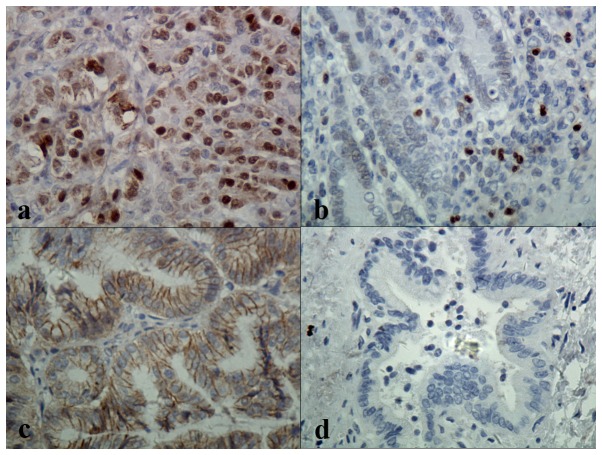
Immunological staining for SFRP1 and β-catenin in human biliary tract carcinoma.

To determine whether SFRP1 and β-catenin expression may correlate with the occurrence of BTC, immunohistochemistry was performed. SFRP1 expression was detected primarily in the cytoplasm in the perinuclear region, while β-catenin was detected in nucleus and cytomembrane (Fig. 1). According to our rating criteria, 52 (66.7%) of the 78 carcinoma specimens were categorized as negative for SFRP1 expression and 26 (33.3%) were categorized as positive. In the normal bile duct tissue, the expression of SFRP1 was significantly higher with 11 (30.6%) negative and 25 (69.4%) positive. Conversely, for β-catenin expression 25 (32.1%) of the carcinoma specimens were negative and (67.9%) were positive; whereas for the normal biliary tract specimens 28 (77.8%) were negative and 8 (22.2%) were positive (Table 2). These results suggest that SFRP1 expression is reduced and β-catenin expression is increased in BTC.

**Table 2:** Immunohistochemical Staining of SFRP1 and β-catenin expression in human biliary tract cancer versus normal bile duct tissues.

Biliary tract status	n	SFRP1		β-catenin	
		Negative	Positive	Negative	Positive
Cancerous	78	52 (66.7%)	26 (33%)	25 (32.1%)	53 (67.9%)
Non-cancerous	36	11 (30.6%)	25 (69.4%)	28 (77.8%)	8 (22.2%)

### SFRP1 and β-catenin expression correlates with clinicopathological features of BTC

To determine whether the expression of SFRP1 and β-catenin correlate with clinicopathological tumor parameters among the 78 BTC samples, we performed chi-square tests. Statistically significant differences in histological type, lymph node invasion and overall survival rate (P<0.05) were observed for both genes, while the TNM stage was only statistically different for β-catenin, and the differentiation grade and occurrence of metastasis after surgery showed no statistically significant difference (Table 1). These results suggest that the reduced SFRP1 expression and increased β-catenin expression in BTC correlate with the status of the disease.

### SFRP1 and β-catenin expression are inversely correlated in BTC

To determine the relationship between the expression of SFRP1 and β-catenin, the 78 BTC samples were further divided into four groups according to expression levels (-, +, ++, and +++). Based on Spearman rank analysis, an inverse correlation between SFRP1 and β-catenin expression was identified (r = −0.636, P<0.0001, Table 3). These results further verify the inverse association of high SFRP1 expression and low β-catenin expression in BTC.

**Table 3:** Inverse correlation between SFRP1 expression and β-catenin expression in human biliary tract cancer*.

SFRP1	β-catenin				Total
	−	+	++	+++	
−	0	3	14	17	34
+	0	2	6	10	18
++	6	4	3	1	14
+++	8	2	2	0	12
Total	14	11	25	28	78

*r = 0.636, *P* = 0.001.

### Univariate and multivariate analyses of survival rates in BTC patients

**Figure 2:**
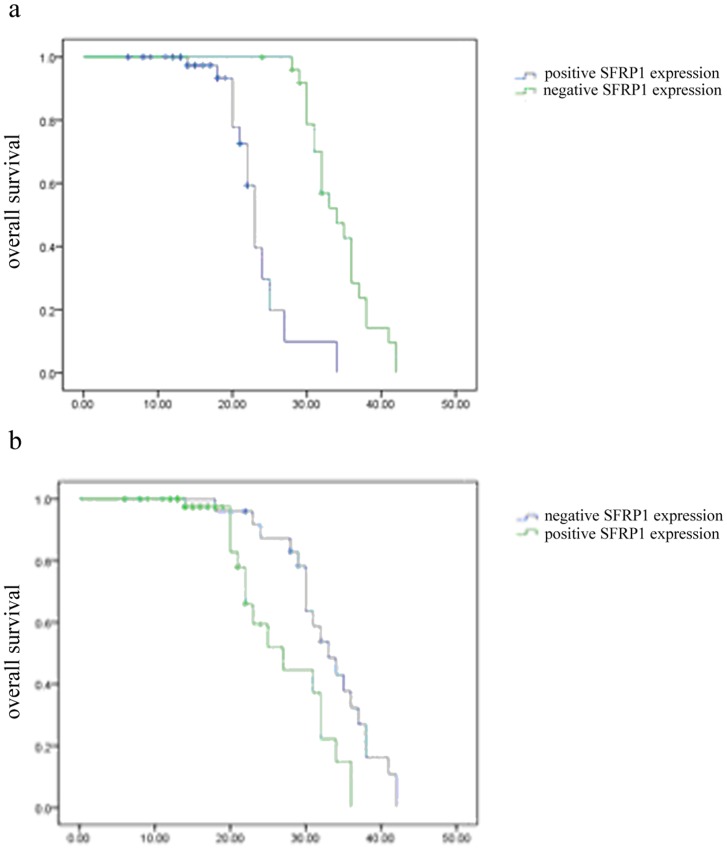
Meier-Kaplan curves of the correlation of SFRP1 and β-catenin expression with overall survival in patients with BTC.

To further examine whether SFRP1 and β-catenin expression correlates with overall survival in BTC, survival curves were calculated using the Kaplan-Meier method. Results indicate that patients with negative SFRP1 expression have a significantly poorer overall survival rate than those with positive SFRP1 expression (P<0.0001, Fig. 2A); and conversely, patients with positive β-catenin expression have a poorer overall survival rate than those with negative β-catenin expression (p = 0.007, Fig. 2B).

To assess the contribution of clinicopathological features as potential prognostic factors, 13 clinicopathologic variables were analyzed in the 78 BTC patients by univariate analysis. The results show that SFRP1 expression, β-catenin expression and histological type are statistically significant factors (Table 4). To further investigate the contribution of these 3 factors we performed multivariate analysis. Only SFRP1 expression could be verified as an independent prognostic marker (Table 5), suggesting that among these variables, high SFRP1 expression may serve as the best prognostic indicator of poor survival in BTC.

**Table 4:** Univariate analysis of clinicopathologic features for overall survival of 78 patients with biliary tract cancer.

Characteristics	n	Survival rate (%)	*P*
**Gender**			
Male	34	54.3	0.693
Female	44	53.5	
**Age(year)**			
<60	32	41.9	0.984
≥60	46	61.7	
**TNM stage**			
I	10	54.5	0.365
II	24	58.3	
III	16	43.8	
IVa	28	55.6	
**Histological type**			
Adenocarcinoma	70	52.9	**<0.05**
Mcinous adenocarcinoma	5	80.0	
Papillary carcinoma	3	33.3	
**Anatomical distribution**			
Intrahepatic	20	30.0	0.267
Perihilar	31	46.9	
Distal	27	65.4	
**Lymph node invasion**			
Present	47	54.7	0.494
Absent	31	52.0	
**Differentiation**			
Well/moderately	36	45.9	0.068
Poorly/undifferentiated	42	61.0	
**Metastasis after surgery**			
+	44	52.3	0.191
−	34	55.9	
**Serum CEA level**			
>5 ng/ml	45	47.2	0.699
≤5 ng/ml	33	59.5	
**Serum CA19-9 level**			
>37 U/ml	48	52.1	0.312
≤37 U/ml	30	56.7	
HBV infection			
+	40	50.0	0.249
−	38	57.0	
**SFRP1 expression**			
Negative	52	73.1	**<0.0001**
Positive	26	15.4	
**β-catenin expression**			
Negative	25	20	**<0.01**
Positive	53	69.8	

**Table 5:** Multivariate Cox regression analysis of prognostic markers in 78 patients with biliary tract cancer.

Factors	Category	X^2^	P	HR*	95% CI*
Histological type	Adenocarcinoma/Papillary carcinoma	0.004	0.951	0.954	0.209–4.358
	Mucoid carcinoma/Papillary carcinoma	0.029	0.866	1.174	0.182–7.558
Lymph node invasion	Absent	0.597	0.440	0.755	0.370–1.540
	Present				
SFRP1 expression	Positive	12.351	<0.0001	10.514	2.831–39.048
	Negative				
β-catenin expression	Negative	2.512	0.113	0.416	0.141–1.231
	Positive				

*HR hazard ratio, CI confidence interval.

## Discussion

BTC is a rapidly progressive disease with a poor prognosis and an emerging trend of increased incidence [27]–[29]. Patients with BTC have no obvious early symptoms due to its anatomical and physiological characteristics and therefore are usually diagnosed at an advanced stage. Though advances have been made in the understanding of BTC, the treatment of this lethal disease is still unsatisfactory. Thus, finding prognostic indicators is important both to improve early diagnosis and to ameliorate the prognosis for BTC.

Aberrant activity of the Wnt signaling pathway has been observed in a variety of human cancers [16]–[24]. Aberrant activation of the Wnt pathway results in the stabilization and/or altered localization of β-catenin, which presumably promotes tumorigenesis by exacerbating the transcription of growth-controlling genes [10]–[12]. Altered localization of β-catenin is a hallmark of Wnt signaling pathway and serves as a marker of pathway activation [14]. In our study, positive β-catenin expression was observed in 67.9% of BTC samples, with only 22.2% positive expression in normal biliary tract specimens. We demonstrate that β-catenin expression in BTC is also markedly related to histological type, lymph node invasion and TNM stage. Patients with positive β-catenin expression have a significantly poorer overall survival rate. These results are consistent with studies of other cancers in which β-catenin expression is associated with unfavourable prognosis [13]–[15] and suggest that the abnormal activation of the Wnt signaling pathway in BTC participates in the progression of the disease.

The putative role of the Wnt signaling pathway in BTC is further supported by immunohistochemical staining of SFRP1, an inhibitor of Wnt signaling [30]. Our study suggested that SFRP1 localization in BTC is predominately cytoplasmic perinuclear, consistent with a previous study of bladder cancer [31]; however, the localization appears uniformly cytoplasmic in immunohistochemical staining of several other tissues in other studies[32]–[33]. The reason for this difference is not known, but could be related to the tissue-specific expression of alternate RP-interacting proteins. Silencing of SFRP1 in cancer has been widely reported [18]–[25], [34], indicating that aberrant inactivation of SFRP1 might be a common mechanism to activate Wnt signaling in solid tumours. We observed that SFRP1expression was reduced in about 70% of BTC specimens, and its expression showed a clear inverse correlation with that of β-catenin expression by Spearman rank correlation analysis. Moreover, SFRP1 expression was significantly associated with histological type and lymph node invasion. Most importantly, patients with negative SFRP1 expression were shown to have a significantly poor overall survival rate as assessed by univariate analysis, and SFRP1 was verified as an independent prognostic factor by multivariate analysis using the Cox proportional hazard regression model.

The molecular cause of SFRP1 loss has not been elucidated; however, epigenetic silencing of gene regulatory regions may be one of the underlying causes for the dysregulation of the Wnt signaling pathway [34]. Recently, a series of studies have confirmed that promoter hypermethylation mediates SFRP1 gene silencing. Related mechanisms have been described in colon cancer [35]–[37], ovarian cancer [18], bladder cancer [38], human mesothelioma [20], prostate cancer [24], lung cancer [39] and most recently, cholangiocarcinoma [40]–[41]. Interestingly, a direct link exists between the epigenetic inactivation of SFRP1 and the overexpression of microRNA (miR)-31, with exogenous overexpression of miR-31 leading to repression of SFRP1 in lung cancer cells [42]. Several other miRNAs are associated with the Wnt/β-catenin signaling pathways in cancer development [43], and it will be of interest to determine whether these miRNAs also correlate with SFRP1 expression and whether miR-31 may regulate SFRP1 or vice versa.
